# Diagnostic imaging of retinal astrocytomas: A case-series

**DOI:** 10.1038/s41433-022-02275-0

**Published:** 2022-10-12

**Authors:** Keri McLean, Rumana Hussain, Heinrich Heimann

**Affiliations:** 1 Liverpool Ocular Oncology Centre, Liverpool University Hospitals Trust, Liverpool, UK; 2 Department of Eye and Vision Science, Institute of Life Course and Medical Science, University of Liverpool, Liverpool, UK; 3 Liverpool Ocular Oncology Research Group, Department of Molecular and Clinical Cancer Medicine, University of Liverpool, Liverpool, UK

**Keywords:** Medical imaging, Eye cancer

## Abstract

**Objectives:**

To use multimodal imaging techniques to characterise features of retinal astrocytomas (RA) which would aid practitioners distinguish them from other causes of non-pigmented fundal lesions.

**Methods:**

Retrospective analysis of notes and imaging of 17 patients diagnosed with RA at a single centre between January 2012 and June 2021 was conducted. Demographics, examination findings and imaging including colour fundus photography, optical coherence tomography (OCT), infra-red (IR) and ultrasound (US) were analysed. These were compared to differential diagnoses, including retinoblastomas, amelanotic choroidal melanomas, choroidal metastases and idiopathic scleromas.

**Results:**

Fourteen patients (82%; 14/17) had idiopathic RA and three (18%; 3/17) were associated with tuberous sclerosis. Mean age at presentation was 43 years. Twelve patients (71%; 12/17) were asymptomatic. Thirteen (76%; 13/17) had better than 6/12 vision, with 41% (7/17) better than 6/6. All lesions were creamy-white. There were two distinct appearances, seven (39%; 7/18) were poorly-defined translucent retinal elevations and eleven (61%; 11/18) were well-defined solid opaque retinal masses. Six (33%; 6/18) displayed clustered, calcified spherules giving them the pathognomonic ‘mulberry-like’ appearance. On OCT, all appeared as dome-shaped retinal thickening with disruption of the inner retinal layers and nine (60%; 9/15) had intra-retinal cystic spaces giving a ‘moth-eaten’ appearance. Mean basal diameter and thickness on OCT was 2.93 mm and 0.86 mm, respectively. High internal reflectivity on US was noted in 92% (11/12).

**Conclusions:**

RAs display characteristic clinical, demographic and imaging features which can aid differentiating them from other non-pigmented fundal lesions. We advise using multiple imaging modalities when diagnosing these lesions.

## Introduction

Retinal astrocytomas (RA) are rare tumours which arise from retinal glial cells [1]. Most patients are asymptomatic, therefore RA are usually discovered as an incidental finding [1]. They are the best-known ocular manifestation of tuberous sclerosis (TS) but can be idiopathic or associated with neurofibromatosis [1, 2]. RA are considered relatively quiescent lesions with little potential for progression, however in rare cases they may exhibit aggressive behaviours including rapid growth, exudative retinal detachments and neovascular glaucoma [1–4].

RA are usually seen as a sessile or slightly elevated lesion in the nerve fibre layer of the retina, but they can have several clinical variations - they can be unilateral or bilateral, solitary or multifocal, transparent or opaque and calcified or non-calcified [1, 2]. Other diseases such as choroidal osteoma, chorioretinitis, amelanotic melanoma and retinoblastomas can have similar clinical presentations. Whilst RA are mostly benign lesions and do not require treatment, some of these other conditions require active management. It is therefore important to be able to identify the characteristics of RA.

Alongside a careful history and examination, a wide array of ophthalmic imaging can be utilised to aid diagnosis of RA. Colour fundus photography will provide a time-dated record of the lesion to assess colour, borders, topography, transparency and vascularity. Serial imaging allows monitoring for growth, regression or other changes in the tumour. Fundal autofluorescence (FAF) indicates the metabolic state of the retinal pigmented epithelium and the tumour itself. Spectral domain -optical coherence tomography (SD-OCT) allows cross-sectional visualisation of the retinal and to some extent the choroid. SD-OCT also provides a visual representation of the intrinsic structure of tumours such as whether it is solid, cystic or contains cavities, and if there is subretinal fluid or infiltration of the choroid. Measurements of the tumour can also be taken from an SD-OCT. Ultrasound B-scans (US) allow measurement of the tumour thickness and assessment of reflectivity, posterior shadowing and tumour extension.

In this study, we provide our experience of using these diagnostic imaging techniques to identified RA, analyse the characteristics of RA on each modality and compare these findings with the current literature. We aim to provide an aid which will help practitioners distinguish RAs from other causes of non-pigmented lesion of the fundus, which can sometimes be challenging.

## Methods

We reviewed the notes and imaging of seventeen patients who were clinically diagnosed with RA at the Liverpool Ocular Oncology Centre (LOOC), Liverpool University Hospitals NHS Foundation Trust, between January 2012 and June 2021. Approval for this study was granted by Liverpool University Hospitals Trust and it is adherent to the Declaration of Helsinki.

Ophthalmic examination included best corrected visual acuity (BCVA), slit-lamp biomicroscopy and indirect ophthalmoscopy. Imaging modalities included colour fundus photography, OPTOS ultra-widefield retinal imaging (California, Optos plc, Dunfermline, UK), SD-OCT (Spectralis HRA + OCT, Heidelberg Engineering, Heidelberg, Germany), infra-red (IR) imaging (Spectralis HRA + OCT, Heidelberg Engineering, Heidelberg, Germany) and US (Ellex Inc, Minneapolis, USA).

Statistical analysis was performed using GraphPad Prism version 6.01 for Windows (GraphPad Software, California, USA). This included frequencies, percentages, range, means and standard deviations. Two-tailed paired t-tests were used to compare the basal diameter and tumour thickness as measured on US B-scan compared with OCT measurement.

## Results

Table 1 outlines the demographics, examination findings and multimodal imaging characteristics. Eighteen eyes of 17 patients were clinically diagnosed with RA; ten right eyes (59%; 10/17), six left eyes (35%; 6/17) and one case (6%; 1/17) of bilateral involvement. The majority were solitary lesions, except the bilateral case which had multiple lesions in both retinae. Of the 17 patients, ten were female (59%; 10/17) and seven were male (41%; 7/17). The mean age at presentation was 43 years (range 12–66). Best corrected visual acuity (BCVA) at presentation ranged from 6/3.8 to 6/48 in a patient with learning difficulties and myopia. Seven patients (41%; 7/17) had a BCVA of 6/6 or better, six patients (35%; 6/17) had between 6/7.5–6/12, and four (24%; 4/17) had 6/15 or worse in the index eye. Twelve patients (71%; 12/17) were asymptomatic, one (6%; 1/17) complained of floaters, two (12%; 2/17) reported reduced vision, another reported occasional blurring of their vision and dizziness (6%; 1/17), and one complained of a paracentral scotoma (6%; 1/17).

**Table 1:** Demographics, examination findings and multimodal imaging characteristics of 17 patients diagnosed with Retinal Astrocytoma.

Demographics							Fundoscopy/Colour Photograph									Optical coherence topography					Fundus autoflourescence			Infrared		Ultrasound			
Patient	Sex (M/F)	Age, years	POH	Tuberous Sclerosis	BCVA (Snellen)	Index Eye (R/L/B)	Location	Juxtapapillary	Disc obscuration	Creamy/white colour	Opaque (O)/Translucent (T)	Well-circumscribed	Mulberry appearance	Vascularity	Retinal Traction	Tumour structure	Dome-shaped	Posterior shadowing	Maximum basal diameter (mm)	Maximum thickness (mm)	Well-defined	Hyperflourescence	Hypoflourescence	Hyper-reflective	Hypo-reflective	High internal reflectivity	Max basal diameter (mm)	Max thickness (mm)	Posterior shadowing
1	M	35	Right uveitis	-	6/15	R	IN	-	-	Y	O	Y	Y	-	-	Cystic with cavities	Y	Y	3.51	1.08	Y	Y	-	Y	-	n/a	4.62	1.45	n/a
2	M	61	Left ambylopia	-	6/6	L	IT	Y	0	Y	O	Y	-	-	-	Solid	Y	Y	2.82	0.82	n/a	n/a	-	-	Y	Y	4.18	1.19	n/a
3	M	49	-	-	6/4.8	L	IT	-	0	Y	O	Y	Y	Y	-	Cystic with cavities	Y	Y	4.36	0.95	Y	Y	-	Y	-	Y	6.01	1.31	Y
4	M	58	-	-	6/4.8	R	IT	-	-	Y	T	-	-	-	-	Solid with cysts	Y	-	2.95	0.62	-	-	Y	-	Y	Y	3.94	0.78	-
5	F	39	-	-	6/7.5	R	N	-	-	Y	O	Y	Y	-	-	Solid	Y	-	2.71	0.47	-	-	Y	Y	-	Y	3.3	1.07	-
6	F	52	Right trauma	-	6/15	R	N	Y	30%	Y	T	-	-	-	-	Solid	Y	-	2.27	0.91	-	-	Y	-	Y	Y	3.37	0.9	-
7	F	46	-	-	6/7.5	R	IT	-	-	Y	O	Y	Y	-	-	Cystic with cavities	Y	Y	2.12	1.06	Y	Y	-	Y	-	Y	4.18	2.09	n/a
8	F	49	-	-	6/6	R	ST	-	-	Y	O	Y	-	Y	Y	Solid	Y	Y	2.35	1.41	Y	Y	-	-	Y	Y	-	-	-
9	F	26	-	Y	6/48	L	ST	Y	0	Y	T	-	-	-	-	Solid	Y	-	2.46	0.52	-	-	-	-	Y	n/a	-	-	n/a
10	M	66	Left BRVO IT	-	6/9.5	R	N	-	-	Y	O	Y	-	-	-	Cystic	Y	-	3.21	0.78	-	-	-	-	Y	n/a	-	-	n/a
11	F	46	-	-	6/3.8	L	IN	-	-	Y	O	Y	Y	-	-	Cystic with cavities	Y	Y	2.13	0.99	-	Y	-	Y	-	Y	3.91	1.88	n/a
12	F	57	-	-	6/6	L	Disc	Y	30%	Y	T	-	-	-	-	Solid	Y	-	2.23	0.80	n/a	n/a	n/a	-	Y	n/a		-	n/a
13	M	28	Adult vitelliform dystropy, Left ambylopia	-	6/6	R	ST	Y	0%	Y	T	-	-	-	-	Cystic	Y	-	2.79	0.71	Y	-	Y	-	Y	Y	4.9	1.21	n/a
14	M	54	Right CRVO	-	6/9	R	IT	Y	50%	Y	T	Y	-	-	-	Cystic with cavities	Y	-	5.21	0.68	Y	Y	-	Y	Y	Y	4.53	0.68	-
15	F	34	Right amblyopia	Y	6/9	B	Multiple	Y	0%	Y	T	-	-	-	-	n/a	n/a	n/a	n/a	n/a	-	-	-	-	-	n/a	n/a	n/a	n/a
16	F	12		-	6/9	R	IN	-	-	Y	O	Y	Y	Y	-	n/a	n/a	n/a	n/a	n/a	n/a	n/a	n/a	n/a	n/a	Y	5.0	2.0	n/a
17	F	27		Y	6/30	L	IT	-	-	Y	O	Y	-	Y	-	Solid with cysts	Y	Y	2.87	1.07	Y	Y	-	Y	-	n/a	n/a	n/a	n/a

Of the four patients (24%; 4/17) who reported ‘skin lumps’, one had a definitive diagnosis of TS, another was subsequently investigated for and diagnosed with TS following the RA finding (patient 17), one had previously been investigated but found not to have TS (patient 12) and the other patient had a genetic deletion in Chromosome 17 (patient 16), although the association of this with RA is unknown. Another patient was known to have systemic manifestations of TS without skin signs (patient 9).

Regarding past ocular history, one patient had previous anterior uveitis in the index eye, one had a previous trauma in the index eye, one had amblyopia in the index eye, one had a branch retinal vein occlusion in the contralateral eye, one had a central retinal artery occlusion in the index eye and one had adult vitelliform dystrophy bilaterally. Eleven patients (65%; 11/17) had no previous ophthalmic history. Nine patients (53%; 9/17) had other past medical histories including diabetes, hypertension, ischaemic heart disease, epilepsy, asthma and TS.

Sixteen of the 17 (94%) patients were discharged from LOOC after their first visit for annual follow-up with their local or referring Ophthalmologist. The other patient who presented with a two-month history of visual loss was diagnosed with an aggressive RA. Following treatment with Verteporfin Photodynamic Therapy (PDT) there was rapid regression of the tumour with an excellent visual outcome of 6/7.5 and no recurrence after five-years of follow-up.

### Ophthalmoscopy and fundus photography

**Fig. 1:**
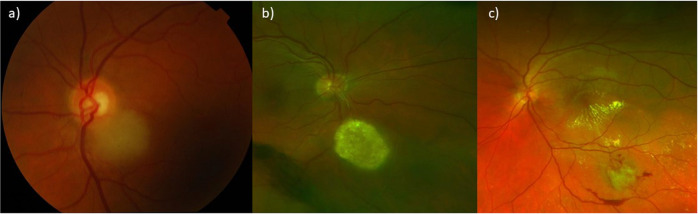
Colour photograph of retinal astrocytomas. **a** RA variant which is creamy, white poorly-defined translucent elevations of the retina, **b** RA variant which is creamy, white solitary, solid, opaque mass with well-defined borders, **c** Aggressive RA with associated haemorrhages and exudates tracking toward the macula.

All lesions had a creamy, white appearance. Two, almost distinct appearances of the retinal astrocytomas were identified. One type, which accounted for 39% (7/18) of eyes, appeared to be poorly-defined translucent elevations of the retina which allowed visualisation of the retinal vasculature (Fig. 1a). The bilateral case with multiple lesions was of this type. The second type appeared to be a solitary, solid, opaque mass with well-defined borders (Fig. 1b). This accounted for 61% for eyes (11/18). Tumour vascularity was noted in four (22%; 4/18) of these lesions, one of which also had retinal traction (6%; 1/18). Additionally, six (33%; 6/18) of these lesions appeared to clustered spherules which formed a mulberry-like shape. One tumour (6%; 1/18) had a mixed appearance with a well-defined small central area of calcification with a surrounding translucent area. One case, patient 17, had associated haemorrhages and exudates tracking toward the macula (Table 1 and Fig. 1c).

Of the sixteen solitary lesions, six (38%; 6/16) were inferotemporal (IT), three (19%; 3/16) were superotemporal (ST), three (19%; 3/16) were inferonasal (IN), three (19%; 3/16) were nasal (N), one (6%; 1/16) was at the optic disc. Seven (44%; 7/16) were juxtapapillary with five (31%; 5/16) lesions obscuring the optic nerve, ranging between 10 and 50% of the disc area.

### Optical coherence topography

**Fig. 2:**
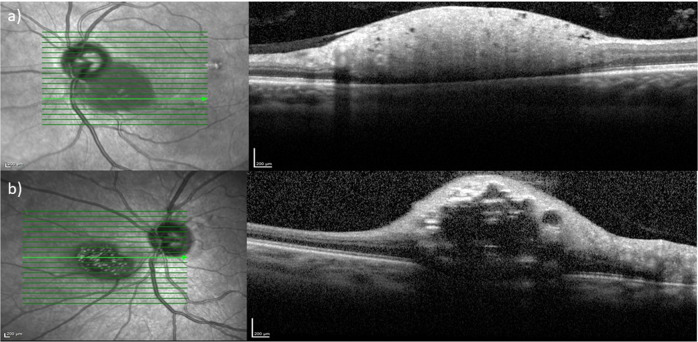
Infra-red (IR) and Optic Coherence Imaging (OCT) of retinal astrocytomas. **a** IR on the left demonstrates well-defined hypo-reflectivity and OCT image on the right demonstrates solid dome-shaped thickening of the inner retinal layers, **b** IR image on the left demonstrates hyper-reflective calcifications with area of hypo-reflectivity and OCT on the right demonstrates dome-shaped thickening of the inner retinal layers, multiple intra-retinal cystic spaces and posterior shadowing.

Optical coherence topography (OCT) through the lesions was performed in fifteen patients. All tumours were confined to the retinal layers and typically appeared as dome-shaped retinal thickening with disruption of the normal layers (Fig. 2). Eight tumours (53%; 8/15) had a solid appearance (Fig. 2a), of which one also had multiple intra-retinal cystic spaces. A total of nine tumours (60%; 9/15) had intra-retinal cystic or cavital spaces which gave them a moth-eaten appearance (Fig. 2b).

Tumour-associated posterior shadowing on the OCT images obscured the choroidal in seven cases (47%; 7/15), however there was no evidence of extension beyond the retinal layers in any of the tumours (Fig. 2b). Two patients (13%; 2/15) were noted to have intra-retinal fluid adjacent to the tumour. OCT-measured maximum basal diameter ranged from 2.12 to 5.21 mm (mean 2.93 mm). Maximum tumour thickness ranged from 0.47 to 1.41 mm (mean 0.86 mm). Computer-generated estimates of the choroidal baseline were used as a surrogate where posterior shadowing on OCT causes partial or complete obscuration of the posterior tumour border.

### Fundal autofluorescence imaging

**Fig. 3:**
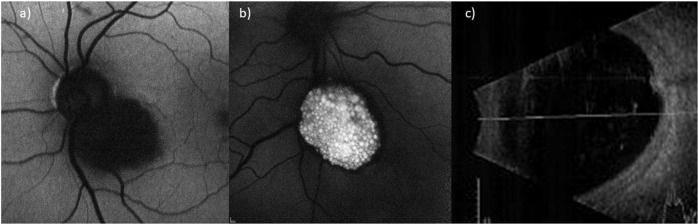
Fundal autofluorescence (FAF) and ultrasound B-scan (US) of retinal astrocytomas. **a** FAF demonstrating hypofluorescence RA. **b** FAF demonstrating hyperfluorescent cystic-like opacities and calcifications giving a mulberry-like appearance. **c** US b-scan of RA demonstrating high-internal reflectivity.

Fundal autofluorescence was available in thirteen cases. Seven (54%; 7/13) were noted to have a well-defined border between tumour and adjacent retina. Five (38%; 5/13) demonstrated hyperfluorescent cystic-like opacities which appeared brighter if they were more superficial (Fig. 3b). Four (31%; 4/13) other tumours has a hypofluorescent appearance (Fig. 3a).

### Infrared imaging

Infrared imaging was available in sixteen cases, six (38%; 6/16) of these had hyper-reflective calcifications with surface calcifications appearing brighter (Fig. 2b). The other nine (56%; 9/16) tumours were hypo-reflective on IR imaging (Fig. 2a).

### Ultrasonography

B-scan ultrasonograpy was available in twelve cases but only ten had measurements documented. Maximum basal diameter ranged from 3.30 to 6.01 mm (mean 4.36 mm) and maximum thickness ranged from 0.68 to 2.09 mm (mean 1.32 mm). High internal reflectivity was noted in eleven (92%; 11/12) cases (Fig. 3c). There was no ultrasound evidence of posterior tumour extension.

In sub-group analysis of ten tumours measured by both OCT and US, there was a mean difference of 1.21 mm in basal diameter which was statistically significant (*p* = 0.001) with larger measurements reports with the ultrasound. There was also a statistically significant mean difference in maximum thickness between the two modalities was 0.43 mm (*p* = 0.004).

## Discussion

Alongside a careful history and examination, diagnostic imaging can provide very valuable information when diagnosing ocular tumours including RA. Frequently, they display distinctive imaging features which can be used to identify them. Sometimes this is from a single modality but more often comparing imaging features across an array of imaging techniques is the most helpful. The majority of these imaging techniques are also quick, non-invasive and available in many ophthalmic units. We have analysed all the available imaging for seventeen of our patients diagnosed with RA. In our experience RA display a number of characteristics and imaging features which can be helpful identifying these tumours from other non-pigmented fundal lesions without invasive biopsy or histology.

### Clinical History and Demographics

Before discussing the imaging features, it is important to take a careful history from a patient presenting with a possible ocular tumour. In keeping with the typical presentation of RA, over 70% of patients in our study were asymptomatic and had been initially identified at a routine optician examination [1]. We found the average age at presentation was in the early forties (mean 43 years old), which is slightly older than the mean age reported in other literature, 33 and 37 years old [5, 6]. Over three-quarters of our patients had better than 6/12 vision at presentation with 41% (7/17) having 6/6 or better. Only three patients had associated TS (17.6%; 3/17). This is lower than other studies which report 48–57% of cases to be associated with TS [5, 6]. We found one bilateral case (6%; 1/17) which is in keeping with the one case found by Semenova et al. in their 25 patient case-series [5].

The clinical history of other differential diagnoses for non-pigmented fundal lesions tend to vary from the above description of RA. Retinoblastoma is sometimes seen as the closest mimic of RA, particularly the aggressive variant of RA. Although curable, it is also fatal if left untreated. It is therefore, probably, the most difficult non-pigment retinal lesion to clinically differentiate from RA. The clinical history and demographics of patients diagnosed with retinoblastoma vary distinctly from RA. A world-wide study of 4351 new retinoblastoma presentations published in 2020, documented the median age at diagnosis was 23.5 months old with a slightly male predominance of 54.6% (2375/4351) [7]. Many patients also had symptoms or signs which prompted referral and diagnosis. Leukocoria was found in 62.8% (2638/4351), strabismus in 10.2% (429/4351) and proptosis in 7.4% (309/4351) [7]. Family history of retinoblastoma was also present in 4.7% (199/4215) [7].

In contrast, choroidal melanomas, of which 15% are amelanotic, tend to occur in an older population, with mean age at presentation, 60 years old [8]. Some choroidal melanoma patients will be asymptomatic but most present with painless loss of vision, metamorphopsia (distortion) or photopsia (flashes) [8, 9]. Another differential, choroidal metastases, are the most common intraocular malignancy and tend to become apparent in the late course of malignancy [10]. Most patients have a known systemic cancer at the time of ocular diagnosis, although studies report up to 35% of patients may not [10, 11]. Of the known primaries, breast cancer is the most common, approximately 40–53% of cases, followed by lung cancer 20–29% of cases [10, 11]. In addition to the different clinical history and demographic, both of these lesions are choroidal, therefore appear different to RAs on examination and imaging.

The demographic of patients diagnosed with idiopathic scleroma is similar to that of RAs. A study of 34 scleroma patients reported all were asymptomatic and the mean age at presentation was 48 years old [12]. These lesions however are distinctly different on examination and imaging because they are scleral in origin.

### Ophthalmoscopy and fundus photography

All of the RA in our study had a creamy-white, amelanotic appearance. In keeping with the literature we found two distinct appearances [1, 5]. The majority (61%; 11/18) appeared as a solitary, solid, opaque mass with well-defined borders and the other 39% (7/18) were a poorly-defined translucent elevation of the retina. A mulberry-like appearance of clustered, calcified spherules was noted in six patients (33%) which is the same as the 33% found in the literature [5]. Additionally one-quarter of the lesions were noted to have vessels on examination which aligns with the 25% found by Semenova et al. [5]. The most common location was inferotemporal (38%; 6/16) and seven lesions (44%; 7/16) were juxtapapillary. This is slightly lower that the 63% of juxtapapillary lesions found in the literature [5].

On clinical examination retinoblastomas initially present as a round, grey intraretinal lesion [13]. With further growth the tumour assumes an irregular lobulated shape, opaque white or pink colour, and may have calcifications [13]. These calcifications have been described as ‘more dull’ and ‘chalky’ when compared to the ‘glistering yellow’ spherules of calcification seen in RA [1]. Advanced retinoblastomas may be associated with subretinal fluid, neovascularisation, glaucoma and retinal detachment [13]. These features are also seen in the aggressive variant of RAs but not a typical RA.

Choroidal melanomas are mostly are pigmented, but around 15% are amelanotic – i.e similar to the fundal colour or pale [8]. Approximately 75% are dome-shaped, 20% are mushroom-shaped and 5% diffuse [8]. Approximately 94% of choroidal metastases appear as a yellow subretinal mass associated with subretinal fluid (75%) [10, 11]. Both these tumours are choroidal, therefore appear deep to the retina in comparison to RAs which is a retinal lesion. Idiopathic scleromas can also be distinguished from RA by their location which is deep to the retina. Although, similar to RAs, they too have been described as yellow-white or orange lesions on fundoscopy with well-defined borders in 71% of cases [12]. Surface calcifications is not a typical feature of these choroidal or scleral lesions.

### Optical coherence tomography

In our experience SD-OCT is probably one of the most valuable imaging techniques to identify the specific characteristics. From the SD-OCT the transition from normal retinal layers to a dome-shaped retinal thickening and disruption of the normal layer could be identified, mostly the inner retinal layer. This is in keeping with the ‘internal retinal disorganization, and a gradual gently sloping transition from a normal retina into a tumorous retina’ described in an OCT study of RA in 15 eyes [14]. Reasonably accurate measurement of the lesion could be taken, although in a few cases posterior shadowing on OCT meant computer generated estimates of the choroidal baseline had to be used as a surrogate marker of the posterior border. The mean tumour thickness was 0.86 mm which is in keeping with the 0.65–0.8 mm reported in the literature by Semenova et al. [5, 14]. The mean basal diameter of 2.93 mm is also in keeping with the 2.9 mm diameter reported by the same group [5]. Additionally, SD-OCT provided information about the intrinsic composition of the tumour. Over half had a solid appearance which is similar to the 46% reported in another study [5]. 60% (9/15) had a moth-eaten appearance due to the intra-retinal cysts or cavity spaces which is in keeping with the 54–67% reported other studies [5, 14]. We did however note less posterior shadowing, 47% (7/15), compared to another OCT study which reported 93% (14/15) [14]. SD-OCT was also useful to detect subretinal fluid tracking toward the macula in one patient diagnosed with an aggressive RA.

Retinoblastoma has probably the most similar appearance on OCT, as it has been described as an optically dense, highly reflective lesion of disorganised tissue of full-retinal thickness [15]. Posterior shadowing can often be observed [15]. Rarely, intraretinal empty cavities have been observed on OCT in more well-differentiated portions of the tumour [15]. By comparison, 60% (9/15) of the RA we reviewed had intra-retinal cystic or cavital spaces on OCT which gave them a moth-eaten appearance.

Both choroidal melanoma and metastases are seen as lesions deep to the retina on OCT or area of choroidal thickening although retinal oedema, thinning, photoreceptor loss and changes to the retinal pigment epithelial layer have also been described [15]. On OCT although idiopathic scleromas can cause a dome-shaped elevation of the overlying retina, the scleral lesion itself is described as having a lumpy, irregular surface at the border between sclera and choroid [15].

### Fundal autofluorescence and Infrared imaging

FAF was helpful in delineating the borders of tumours. Only half of the tumours had a well-defined border which is similar to the literature [5]. Additionally the cystic-like opacities seen on SD-OCT appearance hyperfluorescent on FAF and even brighter if they were more superficial. This suggests the presence of lipofuscin from degenerating tissue or presence of other fluorophore. Infrared imaging also demonstrated hyper-reflectivity of the cases with a mulberry-like calcified spherule appearance. Over half of the other tumours were hypo-reflective on IR.

Similarly FAF highlights the calcifications of untreated retinoblastomas as bright hyperfluorescent areas [16]. This can be helpful if they are subtle. FAF can be helpful in both choroidal melanomas and metastasis to identify lipofuscin (orange pigment). These appear as bright areas of hyperfluorescence which correlate to lipofuscin seen on fundoscopy; lipofuscin is not a described feature of RA [8, 10]. Yellow-white idiopathic scleromas can usually be identified as hyper-reflective, poorly defined lesions on FAF but the orange lesions are more difficult to identify [12].

### Ultrasonography

The majority of RA which had US available were noted to be hyperechoic (92%; 11/12) which is slightly more than the 54% noted by Semenova et al. although they did document 46% as moderate reflectivity [5]. Given the small size of the RA measuring them on US is challenging. The mean basal diameter was 4.36 mm which was the same as the 4.4 mm mean documented by Semenova et al. [5]. Again the mean thickness was 1.32 mm which was similar to the 1.4 mm reported by the same group but slightly less than the mean of 2.1 mm reported by Shields et al. [5, 14].

Interestingly our ultrasound measurements and those reported by Semenova et al. are larger than that measured on SD-OCT [5]. Paired analysis of ten tumours demonstrate a statistically significant difference between the mean difference measured by both OCT and US in both basal diameter (*p* = 0.001) and tumour thickness (*p* = 0.004). Larger measurements were reported on US. The considerable differences between US and OCT measurement of small lesions had previously been noted in choroidal melanoma studies. Shah et al. report that US over-estimates the measurement of small choroidal melanomas by 126% compared to OCT [17].

Retinoblastomas on US typically appears as an echogenic soft-tissue mass with variable shadowing due to fine calcifications and the heterogeneity caused by necrosis and haemorrhage of the tumour [18, 19]. Choroidal melanomas and metastases are both seen to arise from the choroid on ultrasound. Melanomas are typically described as acoustically hallow on B-scan, whereas metastases are documented to have a hyperechoic appearance on US with a significantly lower height to base ratio than a choroidal melanoma [10, 20].

### Strengths and limitation of the study

This is the largest single-centre study on multi-modal imaging of RA. Strengths of this study include its large size given RA is a rare retinal tumour and the availability of multiple imaging modalities to analysis. Additionally our findings support the limited body of evidence of multimodality imaging of RA [5, 6, 14]. Our study is limited by its retrospective nature which meant some of the imaging modalities may not have been available for each patient, for example fundal autofluorescence and ultrasound imaging was not available for all patients. A prospective study would have aided more data collection. Additionally, this was a single centre-study; collaboration with other centres may have increased the study size.

## Conclusion

In conclusion, RAs display certain clinical and demographic features as well as various identifiable characteristics on multimodal imaging which can aid the practitioner differentiate them from other causes of non-pigmented fundal lesions. We advise considering the clinical history and features of multiple imaging modalities when diagnosing these lesions.

## Summary

### What was known before

- Distinguishing retinal astrocytomas from other causes of non-pigmented fundal lesions is highly important but it can be a challenging clinical diagnosis to make.

### What this study adds

- We compare multi-modal imaging characteristics of retinal astrocytomas with those of other causes of non-pigmented fundal lesions, including retinoblastomas, amelanotic choroidal melanomas, choroidal metastases and idiopathic scleromas.
- We provide an aid to help practitioners distinguish retinal astrocytomas from other causes of non-pigmented lesions of the fundus.

## Data availabilty

The datasets generated during and/or analysed during the current study are available from the corresponding author on reasonable request.
